# A single-nucleotide exon found in *Arabidopsis*

**DOI:** 10.1038/srep18087

**Published:** 2015-12-10

**Authors:** Lei Guo, Chun-Ming Liu

**Affiliations:** 1 Key Laboratory of Plant Molecular Physiology, Institute of Botany, Chinese Academy of Sciences, Beijing 100093, China; 2 Graduate University of the Chinese Academy of Sciences, Beijing 100049, China

## Abstract

The presence of introns in gene-coding regions is one of the most mysterious evolutionary inventions in eukaryotic organisms. It has been proposed that, although sequences involved in intron recognition and splicing are mainly located in introns, exonic sequences also contribute to intron splicing. The smallest constitutively spliced exon known so far has 6 nucleotides, and the smallest alternatively spliced exon has 3 nucleotides. Here we report that the *Anaphase Promoting Complex subunit 11* (*APC11*) gene in *Arabidopsis thaliana* carries a constitutive single-nucleotide exon. *In vivo* transcription and translation assays performed using *APC11-Green Fluorescence Protein* (*GFP*) fusion constructs revealed that intron splicing surrounding the single-nucleotide exon is effective in both *Arabidopsis* and rice. This discovery warrants attention to genome annotations in the future.

Most eukaryotic genes carry protein-coding exons that are separated by non-coding introns^1^,^2^. Pre-mRNA splicing is performed by the spliceosome, a large ribonucleoprotein complex comprised of five small nuclear ribonucleoproteins (snRNPs U1, U2, U4, U5 and U6) and a large number of associated proteins^3^,^4^. The size of introns ranges from 13 to over 300,000 nucleotides^5^,^6^. Sufficient evidence suggest that intronic sequences not only determine the splicing pattern^7^, but also have regulatory functions in gene expression^8^. Although most known regulatory sequences including the conserved GT and AG located at the beginning and the end of introns, respectively, an A at the branch point and a pyrimidine tract in spliceosome-binding and intron splicing are located in introns^9^, exonic sequences play an important role in accurate splicing as well^10^,^11^,^12^. The average size of exons is approximately 130 nucleotides in vertebrates, and 180 nucleotides in plants^13^. Studies have showed that exons with less than 51 nucleotides may cause exon skipping, and exons that are too small in size may hinder the recognition of adjacent spliceosome binding^14^,^15^,^16^,^17^. However, internal micro-exons with less than 25 nucleotides have been identified in different eukaryotic organisms by sequencing and computational analyses^18^,^19^. The smallest naturally available exon that has been experimentally characterized so far has 3 nucleotides^16^,^20^. Here we report the identification of a single-nucleotide exon in *Arabidopsis*.

## Results

### *APC11* cDNA in GenBank is mis-annotated

**Figure 1:**
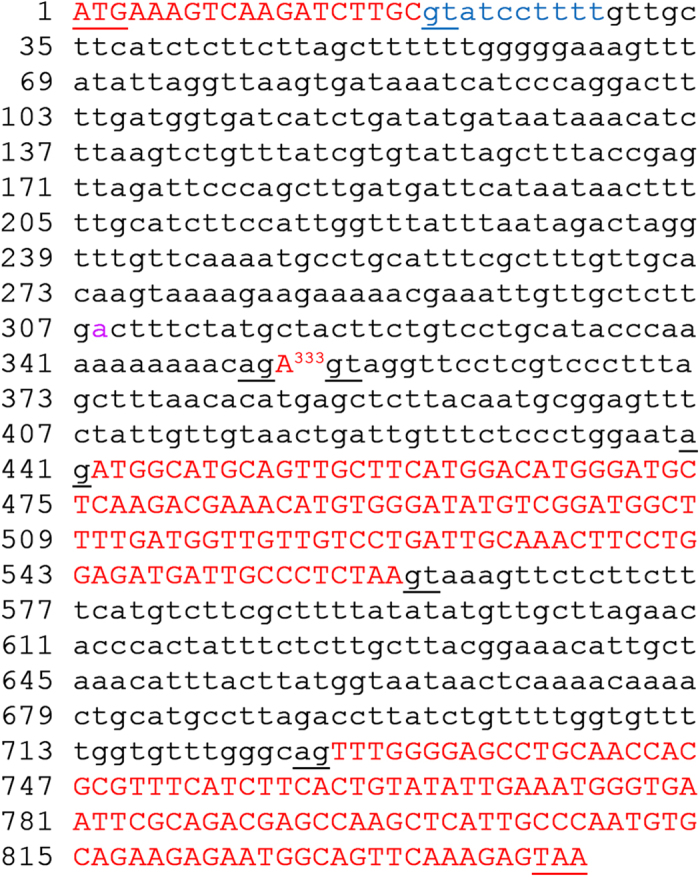
The genomic sequence of *APC11.* The coding region of *APC11*, in which putative exons are highlighted in red and capital, introns are denoted in black and lower case, and the putative branch point “a” is highlighted in purple. A333 is the putative single-nucleotide exon. Conserved intron-exon splicing sequences “gt” and “ag” are underlined and in lower case. Start and stop codons are underlined and in capital. The mis-annotated exonic sequence in GenBank is highlighted in blue.

*APC11* (*At3g05870*) is a single-copy gene in the genome of *Arabidopsis thaliana*^21^. Current annotation predicts that *APC11* has three exons and two introns, and its coding sequence (CDS) contains 261 nucleotides, producing a polypeptide with 87 amino acids (AAs)^21^. However, sequencing of *APC11* cDNA performed in this study has identified only one CDS with 252 nucleotides (highlighted in red; Fig. 1), encoding a polypeptide with 84 AAs. The discrepancy was partially caused by the inclusion of 10 nucleotides from the first intron to the exon in previous annotation (highlighted in blue; Fig. 1).

Further, alignment of the cDNA obtained with the *APC11* genomic sequence revealed a single-nucleotide A inserted into the cDNA. The mysterious A is not in continuity with the CDS in the genomic region. The insertion is absolutely required for in-frame *APC11* translation. Re-sequencing of the *APC11* genomic DNA extracted from both Col-0 and L*er* ecotypes confirmed that the genomic sequence available in the GenBank of National Center for Biotechnology Information (NCBI) is correct, while its cDNA annotated is wrong. We therefore speculate that the extra A may originate from a single-nucleotide exon located in the intron between the previously annotated first and second exons. Within the assigned 422-nucleotide intronic sequence we identified a putative A (designated as A333 in Fig. 1), surrounded by GT and AG, located 333 nucleotides after the upstream exon-intron junction. A putative branch point A was detected 44 nucleotides upstream of the A333 (highlighted in purple; Fig. 1).

### A333 is a functional single-nucleotide exon

**Figure 2:**
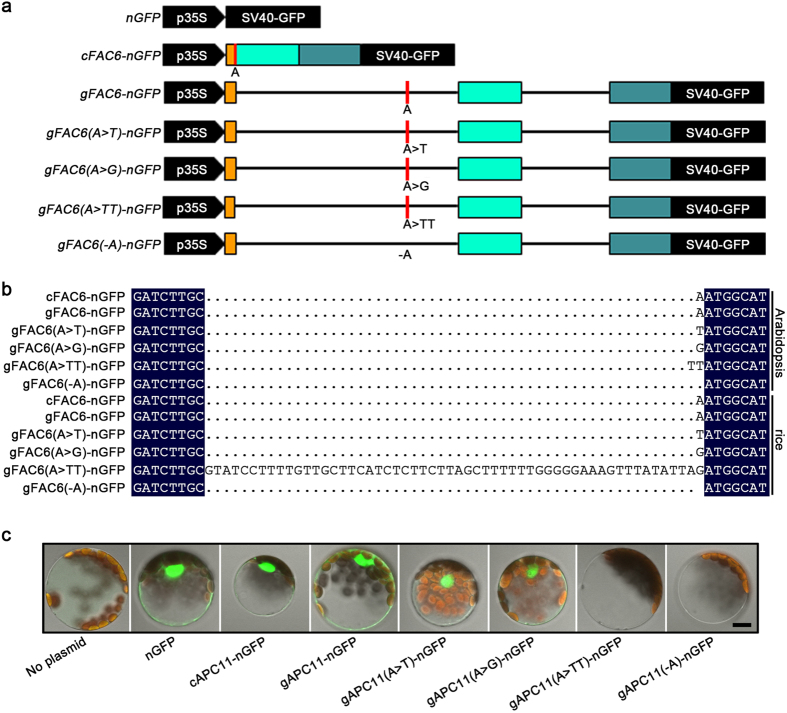
*In vivo* transcriptional and translational assays in *Arabidopsis* and rice protoplasts. (**a**) Constructs generated for transient assays. The CaMV *35S* promoter was used to drive the expression of *APC11* cDNA, genomic or different substitution constructs fused with a nucleus-localized *SV40-GFP* reporter gene. Boxes in orange, cyan and grey indicate three previously identified exons in *APC11*. The black lines indicate introns, and the A333 is shown as red vertical bars. (**b**) Alignment of *APC11* cDNA produced in transgenic *Arabidopsis* or rice protoplasts. Identical nucleotides are shaded. (**c**) Examinations of GFP fluorescence in *Arabidopsis* protoplasts transfected with constructs illustrated in a. Note that GFP signals are detected only in protoplasts transfected with *nGFP*, *cAPC11-nGFP*, *gAPC11-nGFP*, *gAPC11(A* > *T)-nGFP* or *gAPC11(A* > *G)-nGFP*. Scale bar = 10 μm for all photos in c.

To test whether A333 indeed represents a single-nucleotide exon, six constructs with nucleus-localized *APC11-SV40-GFP* fusion proteins expressed under the control of the cauliflower mosaic virus (CaMV) *35S* promoter were made: 1) *gAPC11-nGFP*: the 839-nucleotide *APC11* genomic sequence, with its stop codon deleted, in-frame fused with a *SV40*-*GFP* reporter gene; 2) *cAPC11-nGFP*: a 252-nucleotide *APC11* cDNA, with its stop codon deleted, in-frame fused with the same *SV40*-*GFP*; 3) *gAPC11(A* > *T)-nGFP*: the same as *gAPC11-nGFP* except the A333 was substituted by a T, which is expected to produce a cDNA with T333 if the A333 is indeed a single-nucleotide exon; 4) *gAPC11(A* > *G)-nGFP*: the A333 in *gAPC11-nGFP* was substituted by a G to determine whether nucleotide types affect the splicing; 5) *gAPC11(A* > *TT)-nGFP*: the A333 in *gAPC11-nGFP* was substituted by TT, which shall cause a TT substitution in the *APC11* cDNA and a frame shift in *APC11* translation, leading to disappearance of GFP fluorescence; and 6) *gAPC11(-A)-nGFP*: A333 in *gAPC11-nGFP* was deleted, which shall produce a cDNA without A333, leading to a frame-shift in *APC11* translation and disappearance in GFP fluorescence (Fig. 2a). These constructs were introduced into *A. thaliana* mesophyll protoplasts individually using a polyethylene glycol (PEG)-mediated transfection^22^ for *in vivo* transcriptional and translational assays.

cDNAs were prepared from RNAs extracted from protoplasts transfected with different fusion constructs to examine their splicing patterns. Afterwards, *APC11-nGFP* cDNAs were amplified from individual cDNAs by polymerase chain reaction (PCR) using a forward *APC11* primer and a reverse *GFP* primer (Supplementary Table S1), and sequenced. Results obtained showed that, when either *cAPC11-nGFP* or *gAPC11-nGFP* was used, a sequence identical to *APC11* cDNA was produced. Interestingly, substitutions of A333 by T [*gAPC11(A* > *T)-nGFP*], G [*gAPC11(A* > *G)-nGFP*] or TT [*gAPC11(A* > *TT)-nGFP*] led to T, G or TT substitutions in the cDNA, respectively (Fig. 2b). Further, deletion of A333 made in *gAPC11(-A)-nGFP* led to production of a cDNA without the A.

Detections of GFP fluorescence were used to define the translation of different fusion constructs. When examined under a confocal microscope after twelve-hour incubations, nucleus-localized GFP fluorescence was observed in protoplasts transfected with either *cAPC11-nGFP*, *gAPC11-nGFP*, *gAPC11(A* > *T)-nGFP* or *gAPC11(A* > *G)-nGFP*, suggesting that in-frame GFP translations were achieved in protoplasts transfected with these constructs. In contrast, no GFP fluorescence was detected when either *gAPC11(A* > *TT)-nGFP* or *gAPC11(-A)-nGFP* was used (Fig. 2c), indicating that the substitution of the A333 by TT or deletion of the A333 impaired the translation of these fusion constructs. These results confirmed that A333 in the *APC11* is a functional single-nucleotide exon.

### Splicing of the single-nucleotide exon is mostly conserved in rice

We then addressed whether the processing capability of the single-nucleotide exon is conserved in rice (*Oryza sativa*, var. Zhonghua 11), a remotely related monocotyledonous species. *APC11* in rice has two paralogs, *OsAPC11-1* (*Os03g0302700*) and *OsAPC11-2* (*Os07g0411101*), both of them lack an intron. Protoplasts prepared from 14-day-old etiolated rice seedlings were used to perform *in vitro* transcriptional assay using above-mentioned six constructs (Fig. 2a). Sequencing of *APC11-nGFP* cDNAs amplified from rice protoplasts showed that, when either *cAPC11-nGFP* or *gAPC11-nGFP* was used in transfections, the intact *APC11* cDNA produced from the same splicing patterns as those in *Arabidopsis* protoplasts were detected (Fig. 2b). Similarly, T or G substitutions were detected in cDNA isolated from protoplasts transfected with *gAPC11(A* > *T)-nGFP* or *gAPC11(A* > *G)-nGFP*, respectively (Fig. 2b). A cDNA without A333, and consequently a frame-shift, was detected in protoplasts transfected with *gAPC11(-A)-nGFP*. These results suggest that protoplasts of rice can splice the single-nucleotide exon accurately and effectively as those from *Arabidopsis*. However, it is interesting to note that, when *gAPC11(A* > *TT)-nGFP* was used, the splicing was incorrect. Additional 56 nucleotides from the first intron were incorporated into the cDNA, leading to a frame-shift in the translation of *gAPC11(A* > *TT)-nGFP*, suggesting that the substitution of A333 by TT has caused an altered splicing pattern in rice, which was not observed in *Arabidopsis*.

## Discussion

Pre-mRNA splicing is essential in gene expression in eukaryotic organisms since most of their genes contain multiple copies of non-coding introns interspersed between exons. Precise removal of introns ensures the accurate production of proteins. Exons in pre-mRNA can be spliced either constitutively or alternatively: the former generates a single splicing product across all cell types and developmental stages in which the gene is expressed, and the latter produces a variety of mRNAs by splicing from the same gene in different arrangements to generate protein diversity^9^,^23^. How these intronic sequences are removed effectively and accurately is still largely unknown, given the fact that the sizes of introns and exons varies tremendously^5^,^6^,^13^.

The average size of internal exons in most eukaryotic organisms is from 130 to 180 nucleotides^13^. Although it has been proposed that exons with less than 51 nucleotides may hinder the recognition of adjacent spliceosome binding, causing exon skipping^14^,^15^,^16^,^17^, micro-exons with less than 25 nucleotides have been identified in different eukaryotic organisms by sequencing and computational analyses^18^,^19^. For example, extensive studies have been performed in a 9-nucleotide constitutive micro-exon in the potato intertase gene and a 6-nucleotide constitutive micro-exon from the chicken *cTNT* gene^24^,^25^. The potato invertase gene carries an exon with 9 nucleotides. When 8 of these 9 nucleotides were deleted, the artificial 1-nucleotide exon was skipped in 33% transcripts produced. When this 9-nucleotide exon was replaced by a 6-nucleotide exon from the chicken *cTNT* gene, over 50% of the transcripts produced skipped or mis-spliced the exon^24^. Another recent study in animal and human brains have identified a whole set of genes carrying evolutionally conserved micro-exons, often with the numbers of multiples of three nucleotides, which are involved in modulating interaction domains of neural proteins through alternative splicing^20^. It is plausible that different regulatory mechanisms are implicated in splicing introns flanking a normal exon or a micro-exon.

Three models have been proposed to explain how pre-mRNA splicing is achieved. The “intron definition” model states that, for introns with moderate sizes, the splicing reaction occurs by pairing of the splice sites at two ends of an intron to remove the introns^3^,^7^. The “exon definition” model is proposed to explain the phenomenon that, for short exons separated by a large intervening intron, attaching a 5′ splice site downstream of the second exon in a two-exon splicing substrate greatly enhances the splicing of the upstream intron *in vitro*^3^,^16^. A “recursive splicing” model, which is proposed recently to explain the removal of large introns successively in several steps using intronic ratchet points^26^,^27^,^28^,^29^. In this study, we identified a constitutive single-nucleotide exon in *Arabidopsis*. *In vitro* transcriptional and translational assays performed in protoplasts showed that splicing of introns around this exon can be achieved accurately in both *Arabidopsis* and rice. We also demonstrated that nucleotide types, either purine or pyrimidine, have no effect on splicing of introns around the single-nucleotide exon. Given the fact that spliceosomes are very large in size^30^, it is very unlikely that the exon definition model could be used to explain the splicing of two introns flanking such a single-nucleotide exon. The intron definition model is more plausible, although it is very unlikely that two introns flanking the single-nucleotide exon could be spliced simultaneously. A combined intron definition and recursive splicing model might be applicable to explain the splicing of introns flanking the single-nucleotide exon, to allow two flanking introns to be removed one after another. Consistent with this hypothesis, it has been reported that in the potato invertase gene the splicing of introns surrounding the 9-nucleotide exon occurs recursively in two steps: the second intron was removed before the first one^24^. Further studies are needed to discriminate these possibilities and to identify regulatory sequences involved in intron splicing around the single-nucleotide exon.

In summary, although how widely such single-nucleotide exons are present in eukaryotic genomes remains to be investigated, the discovery of the functional single-nucleotide exon undoubtedly has significant impact on genome annotation in the future.

## Materials and Methods

### Plant materials

*Arabidopsis thaliana* plants (ecotypes Col-0 and L*er*) were grown at 21 °C in a growth room with 16 h of light (100 μmol photons m^−2^sec^−1^) per day.

### Constructs

*SV40-GFP* was amplified from *pPLV04*^31^. The full-length *APC11* cDNA (*cAPC11*) was amplified from cDNA prepared from Col-0 seedlings using reverse transcription polymerase chain reaction (RT-PCR), and *APC11* genomic DNA (*gAPC11*) was amplified from Col-0 or L*er* genomic DNA. *SV40-GFP* and either *cAPC11* or *gAPC11* were ligated simultaneously into *p326-cGFP* digested with XbaI (NEB, USA) and KpnI (NEB, USA) using a one-step cloning assay^32^ to produce *p35S:cAPC11-nGFP* or *p35S:gAPC11-nGFP*, respectively. To generate *p35S:nGFP*, *p35S:gAPC11-nGFP* was digested by XbaI and ligated with T4 DNA ligase (NEB, USA). For A333 substitutions, point mutations were introduced to *p35S:gAPC11-nGFP* using the primers listed in Supplementary Table S1 to produce *APC11*(*A* > *T*), *APC11*(*A* > *G*), *APC11*(*A* > *TT*) or *APC11*(-*A*).

### Protoplast transfection

For protoplast transient expressions, well-expanded leaves from 4-week-old *Arabidopsis* plants (Col-0) were chosen, and the assays were performed as previously described^22^. For protoplast transient expression in rice, seeds (*Oryza sativa*, var. Zhonghua 11) were germinated on half-strength MS basal salts medium and cultured in the dark at 26 °C for 10 to 12 days before protoplasts were isolated and assays were performed as in *Arabidopsis* except the Macerozyme R-10 was replaced by Macerozyme RS (Yakult, Japan).

### Microscopic analyses

To examine the expression of *GFP* in transfected protoplasts, a confocal laser scanning microscope (FV1000MPE, Olympus, Japan) equipped with 488 nm excitation laser was used.

### RNA extraction and RT-PCR

Total RNA was isolated from transfected *Arabidopsis* or rice protoplasts using the Plant Total RNA Purification Kit (GeneMark, China), reverse-transcribed using the FastQuant RT Kit (TIANGEN, China), and sequenced.

## Additional Information

**How to cite this article**: Guo, L. and Liu, C.M. A single-nucleotide exon found in *Arabidopsis*. *Sci. Rep.* **5**, 18087; doi: 10.1038/srep18087 (2015).

## Supplementary Material

Supplementary Information
