# Supplementary material for: A single-nucleotide exon found in Arabidopsis
Source: Sci Rep. 2015 Dec 10;5:18087. doi: 10.1038/srep18087 (PMC4674806; doi:10.1038/srep18087)
Supplement: Supplementary Information [file srep18087-s1.pdf]

A single-nucleotide exon found in *Arabidopsis*

Lei Guo and Chun-Ming Liu

Primer names	Primer sequences
SV40-GFP	GAATGGCAGTTCAAAGAGTCTAGAATGGCTCCAAAGAAGAAGAG
	CAAATGTTTGAACGATCGGTACCTTACTTGTACAGCTCGTCCATG
APC11	CATTTGGAGAGAACACGTCTAGAATGAAAGTCAAGATCTTGC
	CTCTTCTTCTTTGGAGCCATTCTAGACTCTTTGAACTGCCATTC
APC11(A-T)	CAGTGTAGGTTCCTCGTCCCTTTAG
	CGAGGAACCTACACTGTTTTTTTTTTGGGTATG
APC11(A-G)	CAGGGTAGGTTCCTCGTCCCTTTAG
	CGAGGAACCTACCCTGTTTTTTTTTTGGGTATG
APC11(-A)	CAGGTAGGTTCCTCGTCCCTTTAG
	CGAGGAACCTACCTGTTTTTTTTTTGGGTATG
APC11(A-TT)	CAGTTGTAGGTTCCTCGTCCCTTTAG
	CGAGGAACCTACAACTGTTTTTTTTTTGGGTATG
APC11-F	ATGAAAGTCAAGATCTTGC
SV40-GFP-R	CTTGCCGTAGGTGGCATC

Supplementary Table S1. Primers used in this study.
